# Being in a position to know: attuned responsiveness as the hallmark of experiential knowledge and expertise in mental healthcare

**DOI:** 10.3389/fpsyt.2024.1490489

**Published:** 2025-01-13

**Authors:** Roy Dings, Derek W. Strijbos

**Affiliations:** ^1^ Faculty of Philosophy, Theology and Religious Studies, Radboud University, Nijmegen, Netherlands; ^2^ Department of Psychiatry, University Medical Center Utrecht, Utrecht, Netherlands; ^3^ Lemontree Interdisciplinary Center for Psychiatry, Psychotherapy and Philosophy, Amsterdam University Medical Center, Amsterdam, Netherlands; ^4^ Dimence Institute for Specialized Mental Health Care, Zwolle, Netherlands

**Keywords:** experiential knowledge, experiential expertise, expertise-by-experience, lived experience (of the illness), lived experience, phenomenology, responsiveness, attunement

## Abstract

Inclusion of people with lived experience into various mental healthcare settings is rapidly increasing. In this article we explicate and address two challenges that hinder this development. First, a descriptive challenge: what is the unique and complementary epistemic contribution of people with lived experience in mental healthcare, precisely? Second, a normative challenge: how to evaluate these contributions of people with lived experience to mental healthcare? To address these challenges, we propose a novel conceptual ‘lens’ through which to understand the epistemic contributions of people with lived experience. Our approach diverges from the orthodox view by not construing these contributions in terms of (experiential) knowledge, but in terms of perceptual and agential skills which put people with lived experience in a unique ‘position to know’. More specifically, we reckon that such contributions are best understood in terms of what we call ‘responsiveness’ and ‘attunement’. The main goal of this paper is to show how the ‘Attuned Responsiveness’-Framework allows us to satisfactorily address the descriptive and normative challenge, thereby providing the practice of expertise-by-experience in mental healthcare with a more solid conceptual basis.

## 1 Introduction

People with lived experience are increasingly involved in (mental) healthcare practice and research.^1^ For instance, they are involved in the co-design of policy, contribute to care organization and the development of clinical guidelines, they organize anti-stigma events, and provide direct care by assisting in recovery and offering practical and emotional support (1, 2). Following this development, researchers are investigating the added value of this inclusion across the full breadth of mental healthcare, including domains such as peer support and recovery (3), innovation and policy (4), treatment protocols (5) and research and funding (6).

Within this literature on the potential roles and purposes of expertise-by-experience in mental healthcare, however, two substantial challenges remain. First, a *descriptive* challenge: what is the unique and complementary epistemic contribution of people with lived experience in mental healthcare, *precisely*? We add the emphasis because their added value is often assumed (and rightly so, we believe), but seldomly specified properly. Second, a *normative* challenge: how to evaluate this contribution of people with lived experience in various mental settings? For instance, in what contexts are contributions based on lived experience (in)apt or (ir)relevant, and how to resolve potential conflicts between claims derived from experiential knowledge and other forms of knowledge?

In this paper we propose a framework that may serve as a conceptual starting point for addressing these challenges, thereby providing a more solid foundation for the practice of expertise-by-experience and involvement of people with lived experience in mental healthcare. Rather than providing a definitive and fully fleshed out model of how expertise-by-experience works and how to evaluate it, this framework aims to offer a conceptual ‘lens’ through which to analyze this practice, so as to further our understanding. In a nutshell, our proposal is this: in virtue of their lived experience, experts-by-experience are particularly well-placed (in comparison to other healthcare professionals) to perceive what matters to people experiencing mental health problems. That is: experts-by-experience are in a unique position to know what appears as important in a patient’s lifeworld.^2^ Thus, we resist traditional analyses of experiential knowledge in terms of ‘knowledge of one’s own lived experience’ – private knowledge that subsequently needs to be transformed to yield expertise that can be used in the service of helping others. Rather, we put emphasis on the perceptual and agential nature of expertise-by-experience and flesh out the role of lived experience in terms of the ability to put oneself in a position to know what is important for the other person. We articulate this ability in terms of attuned responsiveness. Hence we propose an ‘attuned responsiveness (AR-) framework’.

In Section 2 we explain the descriptive and normative challenge and show how existing accounts neglect or fail to meet them. Sections 3 and 4 outline our proposal, most notably its key concepts (responsiveness and attunement); Section 5 clarifies how the AR- framework addresses the descriptive and normative challenge; Section 6 offers some ideas on how to further flesh out the proposed framework by relating it to ideas from phenomenology and enactive cognitive science.^3^

To preface our proposal, we wish to highlight two things. First, that the practice of expertise-by-experience is complex and that no one-size-fits-all account should be expected (1, 2). For that reason, we think a pluralistic approach to expertise-by-experience is most promising (7). That is, it does not make sense to treat experiential knowledge and expertise as a homogenous phenomenon. Instead, we think that what is meant by experiential knowledge and expertise actually consists of a variety of forms of knowledge and practical exchanges. This, in turn, informs our current endeavor and adds a fair amount of modesty. We zoom in on what we believe to be a central aspect of expertise-by-experience (attuned responsiveness) which is often hinted at, but has not yet been clearly explicated. We do not want to suppose that our framework offers a complete account of expertise-by-experience, however. There is more to the practice of expertise-by-experience than attuned responsiveness.

A second point we want to make in advance is that in order to understand the added value of including people with lived experience in mental healthcare, we may discern political-ethical from epistemological motivations (7–9). We do not wish to downplay the political-ethical importance of expertise-by-experience, nor the fact that these motivations may be intertwined (e.g. having experiential knowledge may be important for representing service users or reduce stigma). We merely wish to flag that the epistemological motivation involves an under-investigated element, namely what the *precise* epistemic contribution of people with lived experience is. This is a pressing matter, as many parties within mental healthcare (including professionals, policy-makers but also experts-by-experience themselves) question the contribution of experts-by-experience and consider experiential knowledge to be ‘vague’ (2, 10–12).

## 2 Two challenges: descriptive and normative

The descriptive challenge urges us to clarify what the precise epistemic contribution of people with lived experience in mental healthcare consists in. More specifically: the epistemic contribution in virtue of their lived experience. The orthodox answer – ‘experiential knowledge’ – is widely considered insufficient: many scholars converged on the point that the conceptual basis of experiential knowledge is weak. For instance, Rowland et al. (13), p.76 investigate patient involvement and note that “[t]he conceptual and theoretical underpinnings remain poorly articulated” and that many programs to involve patients rely on policy initiatives for their conceptual basis. Rowland et al. (13) stress that “this lack of conceptual clarity participates in dilemmas of program design, implementation and evaluation”. Castro et al. (2) focus on experiential knowledge and note that “the absence of theoretical and conceptual clarity has led to poor understanding and miscommunication among researchers, health practitioners, and policy makers” [(2), p.307]. They remark that “in literature and practice as well, different understandings by different stakeholders are reported” (ibid.). Indeed, Kiili and Itäpuisto (14) note that no consensus seems to be achieved and that even within the same organization, different experts-by-experience may have different views on what they contribute. Leemeijer (11) conducted a qualitative study on the usage of different kinds of knowledge by teams in mental healthcare, and found that experiential knowledge in particular was neglected because of “uncertainty about the meaning and added value of experiential knowledge” (ibid., p.249). Furthermore, Halloy et al. (15, p.405) found that despite popular and institutional success, the concept [of experiential knowledge] remains loosely defined with the result of weakening its heuristic scope and paving the way for its commodification. The term ‘experiential knowledge’, as it is currently used, is a polysemous, catch-all concept that applies to myriad definitions. In the scientific literature, the concept is rarely defined explicitly or is solely evoked in a broad and imprecise way as ‘knowledge derived from patient experiences’.

To be clear, there is an influential view on expertise-by-experience that was developed by anthropologist Thomasina Borkman from the 1970s onwards (16). It is a threefold framework which suggests that experience may be reflected on and integrated (e.g. into a self-narrative) to become experiential knowledge, which in turn may be transformed into experiential expertise when one gains the skillset to put the experiential knowledge to work, e.g. in assisting others in their recovery. This threefold framework still seems to be the main inspiration for many researchers, policy-makers and experts-by-experience, who either implicitly or explicitly adopt it (2, 5, 17).

However, this framework fails to meet the descriptive challenge.^4^ The worry is, quite simply, that it lacks sufficient clarity and detail. This echoes the worry of Halloy et al. (15) that people talk about experiential knowledge in “broad an imprecise” ways. In fact, we think this goes for all elements in Borkman’s framework, as well as the relation between them. First, Borkman (as well as research and policy relying on Borkman’s framework, e.g. (2, 17) relies on a simplified notion of experience, as something that seems to be ‘given’, homogenous and uncontroversial. Such views neglect that experiences are complex and heterogeneous (20–22). It is not clear which experiences are at stake, nor what it is about those experiences that enables knowledge (19, 23). Second, traditional views on expertise-by-experience stress both the role of experience as well as the role of self-narrations and stories of recovery. But a narration (often based on a memory) of an experience typically does not accurately reflect the original experience (24, 25). Although we agree with the traditional view that both lived experience and narratives are important, we criticize the fact that the traditional model lacks a way to reconcile the two.

Turning to (experiential) knowledge, we run into the same problems. Existing work tends to remain silent on how, exactly, experience leads to knowledge. In fact, it is often merely posited that there is ‘knowledge’ at stake, without any further argument or elaboration. Indeed Halloy et al. (15) noted that ‘definitions’ are often somewhat circular and superficial, as experiential knowledge is simply defined as ‘knowledge derived from experience’. Epistemologically, one would want to know e.g. what type of knowledge this is, whether it is fallible, how it connects to other forms of knowledge, how one obtains it and so forth (26). These epistemological concerns too are addressed only superficially. The process of obtaining experiential knowledge, for instance, is only described in a manner where it is not apparent how one could ‘fail’ to obtain it (7). As for the type of knowledge, scholars often refer to e.g. ‘embodied knowledge’, ‘tacit knowledge’ or ‘practical knowledge’. Yet identifying the knowledge at stake *as* ‘embodied knowledge’, for instance, is not sufficient. (7) concluded that any detailed account of experiential knowledge hinges on a more careful elaboration of what this ‘embodied knowing’ precisely amounts to. On the traditional view it is ambiguous whether everyone with lived experience *also* has experiential knowledge. Furthermore, it is unclear whether experiential knowledge is a gradual phenomenon (where arguably everyone has some experiential knowledge) (23).

The third notion, of expertise, faces the same worry, i.e. that it lacks specificity. Borkman refers to this expertise as the “competence or skill in handling or resolving a problem through the use of one’s own experience” [(16), p.447]. This is not highly informative as to the precise expertise at stake, which means that it is difficult to compare this to other forms of expertise. The precise role of experts-by-experience within mental healthcare is not clearly delineated and often debated (27). For instance, policy and training documents for expertise-by-experience remain silent on this matter or only provide very coarse-grained descriptions of what experts-by-experience might do (cf. 17). The problem points towards the descriptive challenge: it is unclear what lived experience contributes epistemically (11). To illustrate, Roennfeldt and Byrne (28) asked people who are tasked with organizing various aspects of mental healthcare concerning their considerations in involving people with lived experience. What they found was that there was certainly a need for specific designated roles but that it was difficult to delineate these because of the worry that in some sense everyone might be taken to have lived experience. Participants found it unclear whether someone needs to have struggled with mental illness per se, and whether and to what extent people have to be recovered from that in order to contribute as an expert-by-experience. For instance, participant Reginald wondered:

“Does it mean you have to have used a public mental health service, does it mean you just have to have had a diagnosable mental health condition, does it mean you could’ve had 6 or 12 months of really struggling, what actually qualifies you for having lived experience? (ibid., p.890).”

Participant Pippa noted that “it means different things to different people. It was not always an easy task to define what lived experience was in the context of lived experience roles.” (ibid.). These kinds of considerations feed into what should be seen as the proper domain (or tasks) of experts-by-experience. In this respect, consider that Kiili and Itäpuisto [(14) pp.5-6])note that “When using contested knowledge, experts-by-experience should also understand their limits, as professional interventions, decisions, and plans are not their responsibility”. To be clear, there *have* to be limits to what experts-by-experience can meaningfully contribute (just as there are limits to what clinicians and scientists can contribute to mental healthcare).

This ties into the second challenge: the normative challenge. It asks us to provide standards of some sort to evaluate contributions to mental healthcare based on lived experience. This challenge comprises several elements, as there are different domains and dimensions relative to which contributions based on lived experience may be evaluated. For instance, when are such contributions relevant, apt or applicable? And how much lived experience, or which lived experience, is required to be relevant or applicable? Can contributions be ‘better’ or ‘worse’ and if so, how should we determine this? Furthermore, how to resolve conflicts, e.g. when experts-by-experience disagree amongst themselves about a specific contribution, or when other professionals (i.e. those who do not rely on lived experience but e.g. scientific or clinical knowledge) disagree with an expert-by-experience. What can or should an expert-by-experience do – what tasks are suitable for them? And which one’s are not, assuming that lived experience (like other sources of knowledge) has limitations? Finally, how to best train experts-by-experience and assess their level of expertise? All of these questions are currently difficult to answer, which is largely due to the descriptive challenge being unresolved. That is, answering these normative questions seems to require that we have a decent and detailed grasp on what experiential knowledge is, precisely.

To stress the importance of this normative challenge, consider that a recent study on how experts-by-experience view their own practice, found that many experts-by-experience questioned their own contribution because they were not always sure whether it was apt and whether it should be called expertise or knowledge at all (12). That is, many worried whether their own lived experiences could be sufficiently ‘generalizable’.

In previous work, we elucidated this requirement of generalizability, in a way that illustrates how the normative challenge and the descriptive challenge are linked (7). The requirement of generalizability holds that the contribution based on lived experience (i.e. what people tend to call experiential knowledge) should not be merely knowledge about one’s own experience and circumstances. It has to go beyond one’s own personal context, to the extent that their experiential knowledge also sheds light on what e.g. an illness is like for someone else and what might work for them. A person who would solely rely on their own lived experience and simply ‘project’ this onto another person that they are trying to help, would clearly not be a very good expert-by-experience. This demand for generalizability, however, poses some problems. Most notably, it creates a tension with the other requirement of experiential knowledge, namely that it is grounded in (idiosyncratic) lived experience. This tension is due to the fact that increasing generalizability comes at the expense of groundedness in lived experience. That is, increasing generalizability inevitably means abstracting away from the details and concrete factors that were inherent to the original lived experience.

This ties into our earlier remark about the tension between experiences and narratives: narrating an experience may involve zooming out and putting it into a broader perspective, thereby increasing generalizability. And indeed the emphasis on narratives is arguably the most common way of increasing generalizability in the literature on experiential knowledge [e. g. (18)]. However, narratives could not by themselves do the trick, for several reasons (7). One reason mentioned by (7) is that narratives can easily be studied by qualitative researchers, so unless experiential knowledge is also something grounded in lived experience, the person with lived experience becomes superfluous, as someone with experiential knowledge, as soon as the narratives are shared.^5^

A straightforward solution to this dilemma is to find an optimal balance between these requirements (of being grounded in lived experience and degree of generalizability). We will return to this issue in Section 5. For now, the point is that because current accounts, predominantly Borkman’s framework and the research and policy that builds on it, fail to satisfactorily address the descriptive challenge, they are also in a difficult position to address the normative challenge. That is: without a detailed account of how both generalizability as well as groundedness in lived experience may be obtained, it remains unclear how to evaluate the epistemic contributions that experts-by-experience make in virtue of their lived experience.

## 3 Responsiveness

We now introduce the Attuned Responsiveness (AR) Framework, in two steps. Section 3 introduces the concept of responsiveness and explains how this allows us to capture the distinctive epistemic contribution of experts-by-experience (i.e., the descriptive challenge). It starts by acknowledging that experience typically modulates subsequent experiences, i.e., the way we experience or are responsive to information in the world. We think that a central element in why people with lived experience are in a unique position to help others who have had similar experiences, is that they have a distinctive responsiveness profile shaped by their own lived experience. Section 4 introduces the concept of attunement. In order to use one’s own lived experience in the service of helping others, one needs to be able to attune to their particular lifeworld, i.e. their particular responsiveness profile. Such attunement involves taking into account relevant areas and degrees of dissimilarity between one’s own lived experience and the experience of the other. In other words, successful attunement requires being or becoming responsive to the (differences in) the other person’s responsiveness to their situation. By highlighting the complexity and heterogeneity of people’s experiences of their health problems, we see that the likelihood of relevant dissimilarities puts pressure on attunement skills. Evaluating epistemic contributions based on lived experience (i.e., the normative challenge) implies that we be able to assess sufficient similarity of lived experience in conjunction with sufficient competence in attunement skills for the specific task at hand.

We wish to stress that for present purposes and for considerations of space, sections 3 and 4 are geared towards conveying the basic gist of our framework, although we elaborate on these ideas in section 5 and briefly embed them in current philosophical and scientific thinking in Section 6.

### 3.1 Being responsive to the world

At the core of the AR-framework is something trivial: people differ in the way that they experience the world. If a dozen people walk through a shopping mall, their experiences will overlap to a certain degree, but there will also be significant differences.

To illustrate, we can easily imagine that an old man notices a bench in the mall that is overlooked by a busy businesswoman. A young boy may notice the bench, but where the bench matters to the old man as it enables him to sit and rest, the young boy may see the bench as being part of a playful environment. To the restaurant employee the dimly lit alley is where he ought to take the trash after closing, but to a teenage girl the alley is felt as something best avoided. A homeless person may notice the warm air coming out of a vent – something that nobody else in the mall pays attention to.

The trivial element that we are hinting at is that these experiences differ in terms of what those people are responsive to. There may be other ways of phrasing this.^6^ Responsiveness pertains to what matters to people, what the world is like to them, what they notice, what affects them and moves them. It involves what people experience as relevant or meaningful, as attractive or dangerous, as inviting them to act (or not). In the example of the people in the shopping mall, this is what makes each experience different: the individuals differ (sometimes slightly, sometimes significantly) in terms of what they are responsive to.

We cannot be responsive to everything. The world contains an enormous amount of information. So much that we cannot be receptive to it all. What we experience, then, is only a subset of what we could experience. As a result, what I am responsive to – what matters to me – need not be the same as what you are responsive to – what matters to you.

### 3.2 What makes one responsive? motivations, experience and reflection

What determines what someone is responsive to? Imagine that we could make a list of everything that a particular individual is responsive to. Let us call this the person’s responsiveness profile. What makes this responsiveness profile the way it is? For present purposes, we may look at three factors.

The first factor is a person’s motivational economy in the broadest sense. Think here of the various taxonomies that motivational psychology has developed since the days of Maslow’s pyramid. We are not concerned with defending any particular taxonomy (as many are contested in one way or the other). What we need for present purposes is the presumably uncontroversial point that human beings all share more or less the same kinds of basic motivations due to our shared biological make-up and shared enculturation and socialization. For instance, because we are biological beings that need nutrients to survive, everyone is responsive to food in one way or the other. Of course this responsiveness may change (e.g. I am more responsive to food when I am hungry compared to when I just ate) but it seems impossible that food simply does not matter to humans. Similarly, human beings are fundamentally social beings, which means that other people, what they do and say, or don’t do or don’t say, is something that we pay attention to in some or other way.

These biological and cultural motivations provide a very coarse outline of what people may be responsive to. They set the bandwidth, so to speak. But within that bandwidth there is certainly room for variation. Genetic and (neuro)developmental variation, together with differences in (sub)culture and upbringing have impact on people’s responsiveness profile. That brings us to the two remaining factors.

The second factor is, in fact, experience itself. This may sound a bit circular: experience entails responsiveness, yet responsiveness is determined by experience? The point though is that experiences and responsiveness are dynamic, circular processes. This means that my current responsiveness is in part determined by previous experiences (as well as, to a lesser degree, future experiences^7^). To illustrate, I am currently responsive to the phrase “doctor” (in the sense that if someone utters this in a room of people I experience this as potentially self-relevant), due to an earlier experience of having obtained a PhD. I am also responsive to commercials about toys (in the sense that other people may not notice this or pay attention to it) because I have a young daughter and the holiday season is coming up.

A third factor in determining our experience is our reflective attitude towards ourselves and our responsiveness. We may become aware that we are responsive in a certain way and, for whatever reason, decide that we wish to alter some particular responses. For instance, I may find that I have been overly responsive to chocolate cake and decide that I should go on a diet. This decision minimally changes my responsiveness and ideally makes it so that I am not responsive to chocolate cake at all anymore (in the sense that I do not notice it in the supermarket and so do not feel inclined to buy it). Note that this is a pivotal element of typical everyday human agency, where in planning ahead we are essentially trying to make ourselves more or less responsive to future situational elements (cf. 29). Such ‘self-programming’ even makes up an important part of our self-interpretative and self-narrating practices (30).

Thus, a person’s responsiveness profile, roughly, comes about due to their biological and cultural background, and modulation through ongoing experiences and self-reflection. Given that people share fundamental biological traits and often share cultural backgrounds, have had similar experiences (in a shared socio-cultural environment) and may engage in similar processes of reflection, responsiveness profiles may overlap in many respects. At the same time, however, because people differ in their socio-cultural background, their (neurodevelopmentally shaped) experiences and reflective processes, there may also be significant differences in these responsiveness profiles.

#### 3.2.1 Dynamics of responsiveness: short-term and long-term alterations

Regarding the dynamics of responsiveness, note that there may be short-term and long-term alterations in one’s responsiveness profile. An experience of being asked whether you have a pen may make someone briefly responsive to pen-like objects, in the sense that prior to this question, the person would not necessarily notice a pen laying around, but after being asked this, a pen stands out in experience. When the pen is found, one’s responsiveness to pens probably drops again. In comparison, some experiences, such as life-events, almost by definition change one’s responsiveness profile in a substantial and enduring way. Becoming a parent, losing a loved one, getting a new job, are all experiences that impact one’s responsiveness in a variety of ways. Again, the point is fairly trivial: getting a new job means you will start noticing things that you previously did not notice. Different things will become relevant or meaningful. The dynamics of responsiveness may also be affected through reflection: deciding that you’ll take a different route to work at a particular moment means you will alter the situational elements that you become responsive to, but only for a limited amount of time. In contrast, choosing to change careers or to migrate will have long-lasting effects on your responsiveness profile.

### 3.3 Trivial and crucial: the role of embodiment and language

So far, we have been emphasizing that responsiveness is fairly trivial. Its triviality stems from the fact that it is extremely common. Everyone is responsive to some things and features in the world and not to others. Being responsive is not an ‘accomplishment’ – one is inevitably responsive in a particular way, due to certain experiences, one’s biological make-up, socio-cultural background and life choices.

At the same time, responsiveness is crucial in that it is an important feature of agency: everything we do, everything we don’t do, everything we say and everything we don’t say, is tied to our specific responsiveness profile. To illustrate: performing action A starts by noticing several things, e.g. that an action is required, that action A is an option, that action A may fit the situation, et cetera. Again, this is extremely trivial but at the same time very important. Consider the action of “putting one’s hand on someone’s shoulder” in order to comfort them. In this case, you have to notice that the person needs comforting, intuit that comforting this person by means of a gentle touch is an appropriate thing to do, feel that this is the right moment to do so, etc. Although trivial, this example shows how crucial responsiveness is in everyday life and social interaction. For as we may know from personal experience, a brief touch (such as a hand on one’s shoulder) may have an enormous impact on the ‘recipient’ of this action. This example also highlights the important role of embodiment: when we speak of responsiveness in terms of ‘noticing’ or ‘attending to something’, this may give the false impression that this is always a ‘cognitive’ form of responsiveness. But oftentimes it is our body that informs us on what matters. Indeed, we perceive the world via our bodies (31). Emotions, bodily tendencies and other affective states play a major role in the way that we experience the world (i.e. in what we are responsive to).

Furthermore, responsiveness ties into our language capabilities, which is important as most practices of human communication (including those of experts-by-experience) include a linguistic dimension. A major reason why you are able to understand this sentence is because you are responsive to these written characters in the right way. Having learned how to read, having learned the English language, having learned some philosophy perhaps, has made you responsive to these words in the right way, in the sense that the ideas that we are trying to convey actually reach you. Again, this is trivial yet crucial: someone who does not know how to read and/or does not know the English language (i.e. someone who is not responsive to these characters in the right way) will not understand our message. As another example, imagine a conversation between three people, two of whom have previously experienced an LSD-trip. These two people can fairly easily communicate about their shared experiences whereas the third person (who has never experienced an LSD-trip) has only a vague idea what they are talking about. The two people may talk about ‘feeling one with the universe’ but the third person does not really ‘get’ what the others mean by this. The reason why the two people are able to communicate so well is that they are each responsive to the words and metaphors they use in the right way. And the reason why they are responsive in the right way is that they had similar experiences which made them similarly responsive in this respect.

### 3.4 Experiences, mental healthcare and responsiveness

All of this puts us in a position to make it intuitively clear why it may be useful and valuable to include people with lived experience in mental healthcare: they offer a unique contribution because they have a responsiveness profile that is distinctive in comparison to the responsiveness profiles of other parties in healthcare (e.g., professionals, policy makers). It is distinctive because it has been (trans)formed by their lived experience of mental illness.

To illustrate, imagine a person, Susan, who is currently struggling with an eating disorder, severe anxiety and panic attacks. The clinic where she is offered treatment employs experts-by-experience. One of these is Joanna who, like Susan, has struggled with eating disorders and anxiety and has recovered. Joanna, as an expert-by-experience, knows what it is like to be in Susan’s position. That is: she picks up on particular features in their shared environment that Susan is responsive to – features that other professionals tend to overlook. Joanna is in a unique position to make sense of what Susan does, does not do, says or does not say.

For instance, Joanna notices that Susan keeps looking at the books on the shelf and knows (from personal experience) that Susan is trying to distract herself from the psychiatrist who confronts her with her weight – a topic that raises anxiety in Susan. When the psychiatrist tries to motivate Susan to take medication, Joanna knows from personal experience that taking medication is an identity-transforming process. Joanna suggests to talk about medication in these terms, enabling Susan to express her worries. Joanna also notices certain phrases that the clinicians use which do not make sense to Susan (as they also did not make sense to Joanna when she was struggling with her eating problems and anxiety).

This is of course merely a sketch, and a hypothetical one at that. But it offers a different lens for looking at the epistemic contribution of experts-by-experience, relative to other healthcare professionals, and why it requires them to have lived experience. On this picture, each discipline can be characterized as having their own distinctive type of responsiveness profile, shaped by training and experience, giving it a distinctive character or ‘salience distribution’ which allows them to perceive and act on a particular range of features of the situation more readily and aptly. For the psychiatrist, the relevant experience is predominantly medical training and clinical experience, resulting in a responsiveness profile that is particularly sensitive to clinical features of Susan’s situation. For an expert-by-experience like Joanna, it is her lived experience that has played an important role in shaping her responsiveness profile such that she can fulfill a distinctive and complementary role in healthcare. Accordingly, she is particularly sensitive to features of Susan’s situation that have to do with, e.g., experiencing illness and trying to cope with it.

## 4 Attunement

The hypothetical example of Susan and Joanna stresses the similarities between their experiences and responsiveness profiles. These similarities explain why Joanna can easily pick up on certain things that are particularly relevant and meaningful (frightening, stressful, comforting, etc.) to Susan. However, no two lifeworlds are the same, that is, no two people are identical in their responsiveness. To start with, people always follow a unique (spatiotemporal) trajectory and occupy a unique position in the world. No two people will have the exact same engagement with their surroundings. Furthermore, there will always be differences in biological make up, socio-cultural background and upbringing, personal experiences throughout the course of one’s life, particular manifestations, experiences of suffering from mental health problems, etc. – differences that result in dissimilarities in responsiveness profiles. Some dissimilarities may become relevant for experts-by-experience.

### 4.1 Sufficient similarity

For instance, imagine that Susan was not helped by Joanna but by Roger, a male expert-by-experience who draws on his experiences with addiction a few decades ago. The point is that even if they have similar experiences under some coarse level of description (e.g. struggling with mental illness, being in therapy, dealing with stigma), some relevant dissimilarities may appear when we adopt a more fine-grained level of description. Experiencing addiction is not the same as experiencing an eating disorder. Being treated with cognitive behavioral therapy is different from being treated with medication. Being stigmatized for having an eating disorder is not the same as being stigmatized for having addiction problems. And trying to cope with mental illness as a man a few decades ago is not the same as trying to do so as a woman in present times.

The issue that we are hinting at, is that of ‘sufficient similarity’ (7). A central assumption in the practice of experiential knowledge and expertise seems to be that the responsiveness profiles of expert-by-experience and the people they are helping are sufficiently similar in relevant respects. Borkman, in her pioneering article, already noted that the experience that forms the basis for experiential knowledge should be “more or less representative of the experience of others who have the same problem” [(16), p. 446]. This seems intuitive: one can only *really* grasp another person’s experiences, if one’s own experiences are sufficiently similar (32). Crucially however, the field of expertise-by-experience has failed to acknowledge and elaborate, let alone overcome the challenge posed by the complex heterogeneity of people’s experiences of mental illness (as another example of this, see (33). For critical evaluations see (19, 20, 27, 28).

The fact that people’s experiences are often dissimilar has implications for the practice of expertise-by-experience. To show why, we turn to an example from a recent study by Kiili and Itäpuisto (14), who investigated experiential knowledge in child and family services. In one case, an expert-by-experience with a focus on addiction was asked to assist mothers struggling with substance abuse. However, it turned out that “the experience of drug or alcohol abuse is not enough if the expert-by-experience invited by the professionals to discuss and support the service users is not also a parent, or, especially, a woman and mother of young children and thus also of having experienced pregnancy” (p.5). What is expressed here, we think, is that the experience of the expert-by-experience (i.e. of addiction) is not sufficiently similar to the experience of the persons being assisted in this particular context (i.e. of being addicted whilst also being a mother). The fine line in establishing sufficient similarity becomes apparent in another example Kiili and Itäpuisto provide, where “an older expert-by-experience was assigned to work with the young parents of small children in a children’s hospital ward” but where “experiential knowledge was not considered beneficial; on the contrary, the parents felt that the expert-by-experience had ‘very old fashioned and confusing ideas of what it means to be a parent’” (ibid., p.6).

### 4.2 Attunement as a set of basic social skills

The issue of sufficient similarity raises the question how people engage with others when there are relevant dissimilarities in their responsiveness profiles. To answer this question, we introduce the concept of attunement. Attunement in human social interaction indicates the myriad of ways in which people probe and adjust their engagement with one another so as to ‘get along’ fluently, relative to the context and goals of their interaction. For our purposes, we want to highlight two aspects of attunement. First, it is a mode of openness in which one is responsive to someone else’s responsiveness. This typically entails paying attention to what someone else is responsive to, and noticing differences in how the other person is responsive [i.e. being attentive, (34)]. Second, attunement may entail various efforts to increase one’s responsiveness to the other person’s responsiveness (i.e., to what matters to them and what they have in mind) in order to improve the ‘fluency’ of the interaction.

The phenomenon that is targeted here is not something mysterious but rather something quite mundane and ubiquitous. Our deflationary position is that dozens of social skills, may, in principle, be involved in processes of attunement (cf. Section 5). For instance, we may use our imagination, “put ourselves in someone else’s shoes”, we may rely on (self-)reflection, on lay theories or cultural narratives [including ‘collective experiential knowledge’, (18)]. We may rely on various forms of empathy, counterfactual thinking and, importantly, make efforts to listen, look and feel more closely so as to make ourselves more sensitive to subtle cues that disclose something about what the person we are trying to understand is responsive to in their field of experience. In many of these social acts, people essentially aim to reduce what they do not know or which lies outside of their scope of understanding by increasing what they do know or broadening their scope of understanding. That is, they to increase and exploit the ‘common ground’, i.e. the shared responsiveness that people do have.

Note that there are many cases where we already know in advance that the person that we are engaging with does not share our responsiveness profile in relevant respects. A good example is interacting with someone from a completely different culture, for instance when on holiday. If you do not speak their language and are not familiar with their customs, you will likely enter any interaction with an open attitude: trying to gauge someone else’s responsiveness (and possibly modifying yours to match it). Another example: children. We all know that a toddler experiences the world differently: what is unreachable to them is reachable to us; what is scary to them is boring to us; what is meaningless to them is meaningful to us (or vice versa). So what people do when interacting with children is they attune to their (differing) responsiveness profiles by using whatever social and empathic skills they possess.

### 4.3 Attuned responsiveness in mental healthcare

Attunement also plays an important role in interactions with people struggling with mental illness. Here too, most people would not simply assume that their interaction partner has (had) similar experiences (alternatively, they would quickly notice when interactions and verbal exchanges miss their mark). When a depressed person talks to someone who has never experienced depression, miscommunication may follow, in the sense that the message that the depressed person tries to convey is not received in the way that the person intends. The reason for this, as we have argued, is that the interlocutor is not responsive in the right way to the words that the depressed person uses. Some people may leave it at that – but some may also put more effort in attuning themselves to the lifeworld (i.e. responsiveness profile) of the depressed person.

While everyone needs to put an effort to attune to people whose lifeworlds are shaped by illness if they want to help or support them, people with lived experience are, on average and in general, in a better epistemic position to do so. Lived experience provides an advantageous position for the process of attunement in at least three respects. First, we think it is plausible that experiences of illness and disability increase a person’s openness or willingness to be responsive to someone else’s differing responsiveness profile. This ties in to the second element, which is that as the result of having experienced illness or disability firsthand, the person with lived experience has insight into how one’s responsiveness to the world may radically change in the course of illness. A person without lived experience may be unaware that a particular feature or structure of the lifeworld could even change at all (and will therefore fail to attune to it). Third, a person with the relevant lived experience shares ways of being responsive with the person they are trying to understand, precisely because they have had similar experiences. This means that attunement may rely on a relatively extended shared responsiveness profile that may be used as a ‘scaffold’ for finetuning social interaction.

The qualification ‘on average and in general’ in the above paragraph is important. We believe there are many ways to put oneself in the position to attune to the responsiveness profile of people experiencing illness. But we think lived experience is the most straightforward, efficient, and probably most effective route. However, attunement comprises a set of social skills, which allow for a range of levels of competence. A person with lived experience may perform poorly in attuning to someone experiencing illness due to poor attunement skills. At the same time, people without lived experience may compensate for their epistemic disadvantage by seeking new information and making use of highly developed attunement skills. Consider, for example, clinicians who combine their clinical expertise with excellent empathic skills and who have further enriched their understanding by listening to and reading about recovery narratives and the phenomenology of mental illness. As a result, they have improved their epistemic position, are more open to possible structural alterations in lifeworlds and important themes in struggling with illness, while also being able to attune relatively well to the things that matter to their patients. Still, however, reading about experience and perceiving it second- or thirdhand, only brings you so far. Lived experience, we believe, enriches one’s epistemic position with detail, depth and practical significance that is hard or perhaps impossible to achieve in any other way.

Moreover, coming back to the issue of sufficient similarity, the epistemic advantage of people with lived experience depends on the extent to which their lived experience (and the way it has shaped their responsiveness profile) is relevant to the particular situation and task at hand. For example, an expert-by-experience who does not share the ethnic background of the patient, may be in a disadvantageous position to understand the way ethnicity influences the patient’s experience of illness and recovery, relative to the clinician with the same ethnicity. Similar considerations apply to themes of gender, age, religion, socio-economic status, etc. What this shows us, is that sufficient similarity cannot be determined in a principled way. It is context- and task-dependent.

## 5 How the AR-framework addresses the descriptive and normative challenge

In Section 2 we discussed two challenges for the practice of expertise-by-experience in mental healthcare. First, a descriptive challenge: what is the unique and complementary epistemic contribution of people with lived experience in mental healthcare, precisely? Second, a normative challenge: how to evaluate this epistemic contribution? We are now in position to address these challenges.

The AR-framework introduces a different conceptual lens through which to examine the practice of experts-by-experience. Instead of relying on the core concepts of experience, experiential knowledge and expertise like Borkman's (16) model, the AR-framework hinges on the concepts of responsiveness and attunement. Importantly, there is no one-to-one mapping of these concepts: it is not the case that e.g. responsiveness ‘equals’ knowledge or that attunement ‘equals’ expertise. Indeed, the AR-framework moves away from the concept of experiential knowledge.

### 5.1 The descriptive challenge

Our impression is that people often think of experiential knowledge as something mysterious and extraordinary (where some proponents even cherish this mysterious character). In contrast, the AR-framework, by addressing the descriptive challenge, tries to show that what experts-by-experience do is often based on quite ordinary capacities related to responsiveness and attunement. The AR-framework suggests that the distinctive epistemic contribution of experts-by-experience in mental healthcare should quite simply be understood in terms of their distinctive responsiveness profile, shaped by their lived experience. Now, the question is: does this constitute a particular form of experiential knowledge? We believe not. Responsiveness comprises a set of perceptual and agential abilities that allow human beings to interact in a distinctively sensitive way with their environment; this is not, strictly speaking, a qualification of knowledge. Rather, we think responsiveness should be understood as creating conditions for acquiring knowledge. Accordingly, our responsiveness profile puts us in a position to understand others and acquire related knowledge. Lived experience contributes to a distinctive responsiveness profile that is particularly sensitive to the things that might matter (are meaningful, frightening, difficult, painful, supportive, etc.) to people struggling with mental health issues. As we have discussed in the previous section, lived experience is surely not the only way to get oneself into such a position. There are many routes to become sensitive to the responsiveness profiles of people suffering from mental illness. But we think it is the highway.

On our proposal, then, the notion of ‘experiential knowledge’, as often used with reference to Borkman's (16) threefold model, is misleading. It suggests that people 1) have experience, 2) gain knowledge of their own experience (through processes of reflection), which they can subsequently 3) learn to put to use in understanding others, as experts (see e.g., 17). We believe this construal creates all kinds of philosophical difficulties. Not in the least: what to make of the essentially private knowledge in step 2 and how to make it generalizable and applicable to other people in step 3. The concept of responsiveness (profile) highlights the epistemic contribution of experts-by-experience without falling into these difficulties. Experts-by-experience adopt a particular epistemic position amidst other professionals, due to their distinctive responsiveness profile, which gives them an epistemic advantage to get to know particular features of other people’s lifeworlds that have to do with struggling with mental health problems. ‘Experiential knowledge’, if we want to stick to this term, is not to be thought of as something private – it is not knowledge of one’s own lived experience. Rather it is knowledge gained through one’s lived experience – a distinctive combination of know-how and acquaintance that is enacted when the expert-by-experience interacts with other people (within the healthcare system).^8^

Let us now look at the role of experience in the AR-framework. On our proposal, experience matters for expertise-by-experience insofar as experience modulates responsiveness. Thus, if one asks “what is it about lived experience that makes it inherently valuable?”, the answer is that it alters one’s responsiveness in a very direct, substantial and enduring manner, resulting in a particular responsiveness profile that likely bears similarity to the responsiveness profile of others who have had similar experiences. Consequently, insofar as lived experience provides an epistemic advantage, one becomes responsive to similar things as the person that you are trying to help (e.g. you notice what they notice, you ‘speak the same language’ and so forth).

There is also a role for other (‘non-experiential’) modalities in the AR-framework. People may rely on reflection, narration, imagination, memory and other strategies to shape their responsiveness profile in order to attune to another person. This means that the AR-framework can easily accommodate the emphasis that a lot of practitioners put on e.g. self-reflection, sharing narratives and accumulated or ‘collectivized’ forms of experiential knowledge – those are simply tools that an expert-by-experience can yield in the process of putting oneself in a position to be adequately responsive to the other person.

Another issue discussed in Section 2 is that the traditional model lacks an explanation of how expertise-by-experience stresses both experience and (self-)narratives, even though these may be in tension (as narratives modify experiences). The AR-framework does provide a way out here by conceptualizing acts of self-narration as means to shape or enrich one’s responsiveness profile (30), to be exploited in attuning to others. Thus in the AR-framework, we can still safeguard the role of experience (as a core determinant of the responsiveness profile) whilst also show why narratives are important in the practice of expertise-by-experience.

With respect to the nature of the expertise involved in using lived experience for the benefit of others, the AR-framework puts emphasis on skilled attunement. More specifically: expertise-by-experience is the expertise to skillfully use one’s responsiveness profile, shaped by lived experience, to attune to the experience of other people (in order to help or support them).

### 5.2 The normative challenge

This brings us to the normative challenge. The normative challenge, to repeat, is to provide standards to evaluate epistemic contributions of experts-by-experience in mental healthcare based on their lived experience. Within the AR-framework we can further articulate the normative challenge in terms of determining sufficiency, in any particular context, of a) similarity in responsiveness profiles in conjunction with b) appropriate attunement skills. That is: relative to the task at hand, what is the relevant degree of shared responsiveness that, in conjunction with certain standards of competence in attunement skills, is minimally required to effectively understand and interact with the people we are trying to help, support, collaborate with or represent? Notice that assessment of (dis)similarity of responsiveness profiles and levels of competence regarding attunement skills are both crucial. Determining sufficient similarity is important to give substance to the idea that it is in virtue of their lived experience that experts-by-experience are a valuable contribution to the field of expertise in mental healthcare. However, given the heterogeneity of people’s experiences of their health problems and the complex entanglement with their (ethnic, religious, etc.) background and other personal characteristics, the ensuing dissimilarity in experience will always put pressure on attunement skills so as to be properly responsive to those differences.

Along these lines, the AR-framework also sheds light on the question which experiences are supposed to be important and who is supposed to qualify as a (potential) expert-by-experience. In the context of mental healthcare, relevant experience presumably include the experience of having one’s lifeworld being disrupted by mental health problems, trying to cope with its social, occupational and existential consequences, finding oneself in the role of a ‘patient’ and try to cope with that, etc. Of course, there is no principled answer as to what kind of experiences are relevant for any specific task within a particular mental healthcare context. We believe that the only way to assess sufficient similarity in any particular context, is to evaluate the epistemic contribution of someone’s lived experience together with the users of mental healthcare services. The examples of Kiili and Itäpuisto (14) discussed in Section 4 are a case in point.

As to who is qualified as an expert-by-experience, the AR-framework also highlights the importance of a certain degree of competence in attunement skills. Crucially, these involve the ability to become sensitive to dissimilarities in lived experience and using one’s responsiveness profile to attune to these dissimilarities in the process of getting to know the other person’s lifeworld and coming to understand what matters to them. Here too, there are no principled answers available – competence profiles depend in the specific task at hand.

Notice that the AF-framework also allows us to address the dilemma, mentioned in Section 2, of meeting two seemingly opposite requirements of ‘experiential knowledge’, viz. being both grounded in one’s own lived experience and generalizable to other people’s situation. The AR-framework suggest we (dis)solve this dilemma by embracing these opposites and stressing the importance of managing a balance between them. Accordingly, lived experience shapes one’s responsiveness profile and puts oneself in a favorable position to be responsive to certain features and themes in other people’s responsiveness profiles. Collective or shared narratives may be useful here (18). This is the extent to which lived experience is generalizable. At the same time, however, dissimilarities between one’s own and other people’s lived experience will always require the ability to attune to such dissimilarities.

How should we assess the epistemic contribution of experts-by-experience, relative to other ‘epistemic’ parties, such as clinicians or scientists? The AR-framework invites us to characterize clinical and scientific expertise as also having their distinctive responsiveness profile and attunement requirements, shaped by professional training and experience. To illustrate, psychiatrists will be particularly responsive, due to their medical knowledge and clinical experience, to psychopathological patterns in what their patients (don’t) say and (don’t) do. Clinical expertise requires the ability to attune their responsiveness to the specific demands of the clinical situation and the preferences and concerns of their patients (38, 39). It could also be that professionals have themselves had experiences of distress or illness that they rely on in their practice (cf. 3).

Like clinical expertise, expertise-by-experience, is to be understood as a distinctive range of attuned responsiveness. As such, there is no theoretical problem how it could be compatible with and complementary to other forms of expertise.^9^ In practice, potential conflicts should be addressed by taking each other’s responsiveness profile seriously and making clear what specific feature or element the experts in question are picking up in the situation at hand. We would think that in most cases, such an approach will bring more detail and nuance to the overall understanding of the situation and turn conflicting statements into mutual enrichment of each other’s judgments.

Finally, the AR-framework allows us to make some recommendations about selecting suitable tasks for experts-by-experience and their training. Evaluating an expert-by-experience as we have seen, involves assessing sufficient similarity in conjunction with appropriate attunement skills. That is: determining whether they are able to exploit the advantage offered by their responsiveness profile in the process of attuning to other people’s experience, while at the same time keeping an eye on relevant dissimilarities. Suitable tasks for experts-by-experience should likewise be based on weighing the importance, in any particular context, of these interconnected requirements: having appropriate levels of competence in attunement skills, together with the potential epistemic advantages of their distinctive responsiveness profiles.

Importantly, weighing these requirements depends on context. Here, it may be useful to rely on a distinction by Tambuyzer et al. (1) between various levels of analyzing mental healthcare: micro (level of care), meso (level of care organization), macro (level of policy) and meta (level of research). Roughly, on the micro level an expert-by-experience’s contribution is aimed at particular individuals in a local healthcare context. Similarity and attunement skills can be evaluated in relation to the lived experience of (and in co-creation with) particular individuals receiving mental healthcare.

However, the further we ‘move up’ to meso and macro levels, the more generic the requirements become. Evaluating similarity will become centered on certain generic themes that inevitably cut across individual differences in responsiveness profiles. Likewise, assessment of required attunement skills at these levels will focus less on someone’s sensitivity to the particularities (including dissimilarities) of concrete person’s lived experience. Instead, more emphasis will be placed on the ability to abstract away from one’s own lived experience, integrate empirical findings (from qualitative research) and avoid bias. This raises the question to what extent lived experience still provides an epistemic contribution at the meso and macro level (and whether e.g., results of qualitative empirical research into the experience of mental illness may provide better epistemic vantage points). With respect to the meta level of research, requirements become more differentiated. Certain topics and experimental designs (e.g., practical research and qualitative methodologies) arguably benefit more from the epistemic contribution of lived experience than others (e.g., epidemiological research and randomized controlled trials).

To be clear, in practice the most optimal way to evaluate the epistemic contribution of experts-by-experience would be an open and transparent process of dialogue and co-creation, where each stakeholders in mental healthcare -either with or without lived experience- can contribute, as opposed to tasks being designated to only a subset of individuals (19, 26). However, even in those cases, it is still important to be clear on where each party’s epistemic strengths and weaknesses lie [e. g. (10, 41)]. Becoming aware of these strengths and weaknesses should be a core element in the training of experts-by-experience. We would reckon that students be trained in (a) the way in which their lived experience modifies their perceptual and agential capacities, and how this beneficially impacts their ability as an observer and participant in healthcare practice, (b) awareness of and reflection on the various normative issues relating to the requirements of attuned responsiveness and, (c) training in the various strategies that may be employed to facilitate attunement. Our impression is that current training programs either neglect or merely marginally include these elements.^10^

## 6 Embedding the AR-framework

Our proposal in the current paper adopts a level of grain that is more specific than traditional accounts of experiential knowledge and expertise, but is still relatively abstract. This is intentional: our goal here is to convey the general idea of this framework, and we believe an overly technical account riddled with jargon hinders this goal. However, we do want to stress that the AR-framework affords to be elaborated in various ways. Given the limited space available, this inevitably involves a mere sketch. The research we discuss below contains worthwhile avenues to elaborate the AR-framework and sets an agenda for future research.

Although there are many different (theoretical) angles from which to elaborate the AR-framework, we think the most promising one is phenomenologically inspired cognitive science (exemplified by recent developments in enactive strands of cognitive science). The emphasis on phenomenology is important because of the centrality of lived experience in the practice of expertise-by-experience (see (22) for an elaborate analysis on this point). Many phenomenological accounts investigate the human ‘lifeworld’ in terms of what we have called responsiveness, i.e. in terms of what matters to an individual, what the world is like for them, what they notice or are affected by, etc., and have analyzed in detail how changes in embodiment and accompanying changes in the ground structure of experience may affect an individual’s responsiveness [for recent accounts, see e.g. (42, 43)]. The AR-framework is *prima facie* compatible with those accounts and they may therefore be used to refine it (22, 44–46). Note that this has significant methodological advantages as well, as recent decades have seen a surge of influential qualitative research methodologies for investigating the lifeworld (both psychopathological as non-psychopathological cases) [e.g. (42, 47)].

Furthermore, cognitive science inspired by such phenomenological accounts investigates the manner in which such lived experience is brought about. It offers us opportunities to ‘look under the hood’. Attention is directed to meaningful elements in our surroundings, resulting in e.g. perceptual salience and affective sensitivity on a (neuro-)phenomenological level. The recent surge in 4E cognition emphasized how most of our everyday agency consists of unreflective responsiveness to relevant and meaningful action possibilities in our environment [e.g. (25, 48)].^11^

The element of attunement also fits nicely with phenomenology and phenomenologically inspired cognitive science. In particular, it sits well with recent social cognition research which stresses the role of embodied and interactional elements [e. g. (50)]. For instance, the idea of ‘participatory sense-making’ holds that people seek to attune the way they act and perceive the world in order to jointly make sense of the world and each other, and vice versa (51). Also of importance here are recent ideas concerning imagination and the extent to which we might simulate alternative ways of experiencing the world (52). We should also stress Ratcliffe's (45) account of ‘radical empathy’, which is an important inspiration for our AR-framework. According to Ratcliffe, when we seek to understand people who are significantly unlike us (e.g. children or people with mental illness) we may rely on phenomenologically inspired ideas such as ‘bracketing’ elements of our lifeworld. Another excellent example of how the AR-framework may be refined is via the idea of 'explanation-aided understanding' as developed by Kendler and Campbell (53): Kendler and Campbell provide illustrative cases of how developments in cognitive (neuro)science may provide conceptual tools and phenomenological insights that may provide further epistemic advantages for social understanding.

Finally, many studies and accounts of the general phenomenon of expertise converge on the idea that it consists in developing, maintaining and finetuning one’s responsiveness to situational demands. What distinguishes an expert chess player from a novice chess player is that the former immediately notices certain opportunities for moving the chess pieces; opportunities that the novice fails to notice. Becoming an expert means modulating this responsiveness through training, where training oftentimes means experiencing it numerous times so that sensorimotor patterns are altered in response to various demands [e.g., (54), Chapter 4].. Similar analyses have been made with respect to clinical expertise (37–39) as well as more general forms of professional expertise [e. g. (55, 56)]. All of these theories are of course complex, so it requires substantial work to flesh out the precise similarities and differences.

To reiterate, we think phenomenologically inspired cognitive science may be beneficial to refining the ARF, but there may be other useful angles as well. For instance, the idea that people with (radically) different experiential backgrounds may struggle to understand each other has also been investigated in the so-called double empathy problem (57). Elements of that debate may likely feed into elaborating the AR-framework. As another example, in the past decades there has been an increasing call to acknowledge the situated, embodied and enacted nature of knowledge, for instance by feminist and standpoint epistemologies [e.g. (58)]. The AR-framework is *prima facie* compatible with those developments. Theoretical engagement with those epistemologies may be mutually beneficial, in that the AR-framework could be grounded in epistemology more firmly, and in turn could provide a more fine-grained account of the perceptual and agential nature of the enactment of knowledge. Finally, we highly welcome exchanges with ‘adjacent’ accounts, such as Kolb's (59) experiential learning model which was developed for educational contexts. Another noteworthy example here would be the account of ‘Deep Experiential Knowledge’ [DEK, (18)]. On our pluralistic approach to experiential knowledge, such DEK may be an additional component in the diverse set of knowledge held by experts-by-experience, but it may also serve as a tool for attunement: relying on collectivized narratives could be used to develop a shared responsiveness).

## 7 Conclusion

We started this paper by pointing out increasing importance of expertise-by-experience in mental healthcare. We wholeheartedly endorse this development, which means that we think it is critical to resolve the descriptive and normative challenges that we outlined in Section 2. For this purpose, we introduced the Attuned Responsiveness Framework, which provides a novel lens through which to understand the practice of expertise-by-experience. In subsequent sections we outlined how the AR-framework may resolve the two challenges, and embedded the framework in neighboring theories and research that may be used to refine and elaborate it.

## Data Availability

The original contributions presented in the study are included in the article/supplementary material. Further inquiries can be directed to the corresponding author.

## Footnotes

1 Because of our own background and expertise, we focus in this paper on the context of *mental* healthcare, yet we reckon that much of what we have to say here will also be of relevance to (health)care more generally.

2 The term ‘lifeworld’ has its origins in the phenomenological tradition yet is also often used in a more colloquial sense to refer quite simply to the ways in which an individual experiences the world. In Section 3 we elaborate the concept of lifeworld in terms of ‘responsiveness’. In Section 6 we suggest that this fairly generic and non-committal construal of the notion of lifeworld in terms of responsiveness is compatible with, and may be enriched by, ongoing work in phenomenology.

3 In developing our framework, we rely on insights from a variety of philosophical and scientific disciplines (e.g. phenomenology, cognitive science), our own lived experience and training as expert-by-experience (RD), our clinical expertise (DS), assistance of a panel of experts-by-experience during our project, and discussions with various stakeholders during an invitational conference on this framework.

4 For reasons of space we cannot fully elaborate Borkman’s model in this paper, but we have extensively discussed and criticized both her pioneering work as well as more recent revisions [e. g., (18)] elsewhere (7, 19).

5 Note that the requirement of being grounded in lived experience also implies that the study of lived experience, for instance by qualitative researchers and phenomenologists, does not amount to them having experiential knowledge. These studies contribute to knowledge about lived experience, which is taken to be distinct from knowledge gained through experience. However, what knowledge gained through experience is, and how it differs from knowledge about lived experience, is unclear, and is precisely what the descriptive challenge is aimed at.

6 As we will discuss in Section 6, phenomenology in particular may be helpful in offering tools that do justice to subtle nuances in these experiences. For the present purpose of presenting the general gist of our framework, we have chosen to rely on colloquial terms rather than phenomenological jargon.

7 This entails anticipation: if someone has told you that you’ll receive a phone call in the next few minutes, then your responsiveness already shifts (e.g. paying more attention to your phone). In this way, a future experience (of being called) changes current experiences/responsiveness.

8 We think the AR-framework is *prima facie* compatible with other work that labeled experiential knowledge in terms of know-how or acquaintance, and offers potential to elaborate these terms (35, 36). See also (37). For instance, attunement may involve knowing how to navigate differing responsiveness profiles, and lived experience comes with acquaintance in the sense that one has ‘direct’ or unmediated access to this knowledge, e.g. via different modalities (cf. knowing that treatment option A is best versus feeling, on a visceral level even, that option A is best).

9 Many accounts on experiential knowledge also stress its compatibility with other forms of knowledge [e.g. (16, 40)]. However, without a detailed account of what experiential knowledge consists of such claims remain empty.

10 The first author has completed a training for expertise-by-experience and has been a guest lecturer in several training programs. An important protocol for the purposes of training experts-by-experience in the Netherlands (cf. 17) illustrates our point here as well.

11 Importantly, research on such possibilities for action or ‘affordances’ may be a useful lens to understand contexts of health care [see e. g. (49)]. For a recent suggestion to construe expertise-by-experience in terms of affordances, see (19).
